# Supplementary material for: Splice-Junction-Based Mapping of Alternative Isoforms in the Human Proteome
Source: Cell Rep. Author manuscript; Available in PMC 2020 Jan 15. (PMC6961840; doi:10.1016/j.celrep.2019.11.026)
Supplement: 2 [file NIHMS1546469-supplement-2.zip › DF1/PXD006675/PulmonaryVein/PulmonaryVein_21_TPM3_AISEELDHALNDMTSG.pdf]

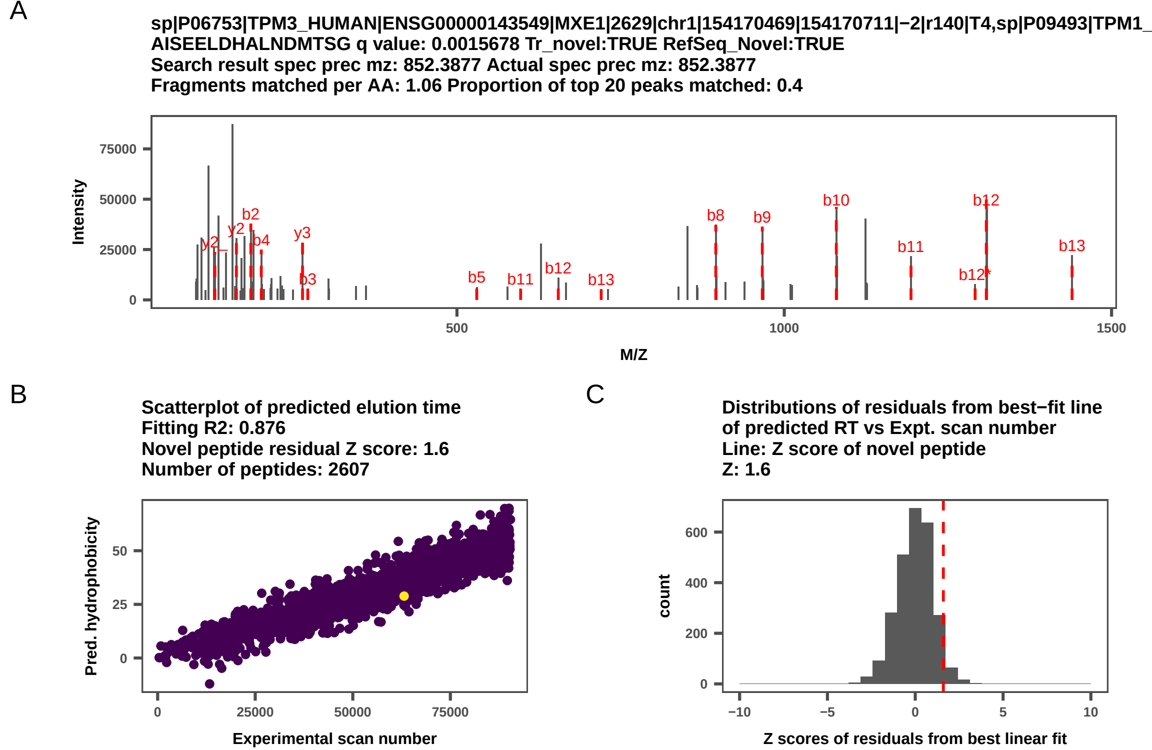
A
sp|P06753|TPM3_HUMAN|ENSG00000143549|MXE1|2629|chr1|154170469|154170711|−2|r140|T4,sp|P09493|TPM1_
AISEELDHALNDMTSG q value: 0.0015678 Tr_novel:TRUE RefSeq_Novel:TRUE
Search result spec prec mz: 852.3877 Actual spec prec mz: 852.3877
Fragments matched per AA: 1.06 Proportion of top 20 peaks matched: 0.4
Intensity
75000
50000
25000
0
y2_
y2
b2
b4
y3
b3
b5
b11
b12
b13
b8
b9
b10
b11
b12*
b12
b13
500
1000
1500
M/Z
B
Scatterplot of predicted elution time
Fitting R2: 0.876
Novel peptide residual Z score: 1.6
Number of peptides: 2607
Pred. hydrophobicity
50
25
0
0
25000
50000
75000
Experimental scan number
C
Distributions of residuals from best−fit line
of predicted RT vs Expt. scan number
Line: Z score of novel peptide
Z: 1.6
count
600
400
200
0
−10
−5
0
5
10
Z scores of residuals from best linear fit
